# Improving survival prediction for melanoma

**DOI:** 10.7554/eLife.48145

**Published:** 2019-06-06

**Authors:** Mykyta Artomov

**Affiliations:** 1 Analytic and Translational Genetics UnitMassachusetts General HospitalBostonUnited States; 2 Broad InstituteCambridgeUnited States

**Keywords:** prognostic biomarker, DNA methylation, cutaneous melanoma, risk stratification, immune-checkpoint blockade, Human

## Abstract

The survival of patients with cutaneous melanoma can be accurately predicted using just four DNA methylation marks.

**Related research article** Guo W, Zhu L, Zhu R, Chen Q, Wang Q, Chen J-Q. 2019. A four-DNA methylation biomarker is a superior predictor of survival of patients with cutaneous melanoma. *eLife* **8**:e44310. doi: 10.7554/eLife.44310

Predicting the risk of outcomes in patients with cancer has traditionally relied on clinical observations: the age of the patient, the size of the tumor, how far it spreads, and how the tumor cells look under the microscope. The accuracy of these clinical evaluations depends on the type of cancer: this approach usually delivers good predictions for cancers that do not spread, but once the cancer metastasizes, the predictive power of this approach declines rapidly.

One of the most challenging cancers to make predictions for is cutaneous melanoma because it progresses rapidly and often spreads into the lymph nodes and other distant organs (Homsi et al., 2005). Cutaneous melanoma is the deadliest skin cancer (Miller and Mihm, 2006), so it is important to be able to manage patient expectations. This means that we need methods other than those based on clinical observations that can predict patient survival.

One alternative approach is based on biomarkers – biological properties within tumors that are associated with melanoma survival. For instance, research showed that several drugs for the treatment of melanoma only targeted tumors that carried a specific mutation in the BRAF gene: the presence of this mutation in a patient is therefore associated with a higher chance of survival due to a positive drug response (Figure 1). Indeed, subsequent research has shown that the higher the mutational 'burden' in the melanoma, the better the response to treatment (Goodman et al., 2017; Figure 1). The interaction between the transcription of genes in the tumor and the immune system is also important: depending on the melanoma tumor type, low levels of transcription of a gene called *MITF* results in fewer immune cells being attracted to the tumor, which leads to an acceleration in tumor growth (Wiedemann et al., 2019). Taken together, these findings highlight that understanding the biological characteristics of melanoma tumors is critical for predicting outcomes and developing new treatments.

**Figure 1.**
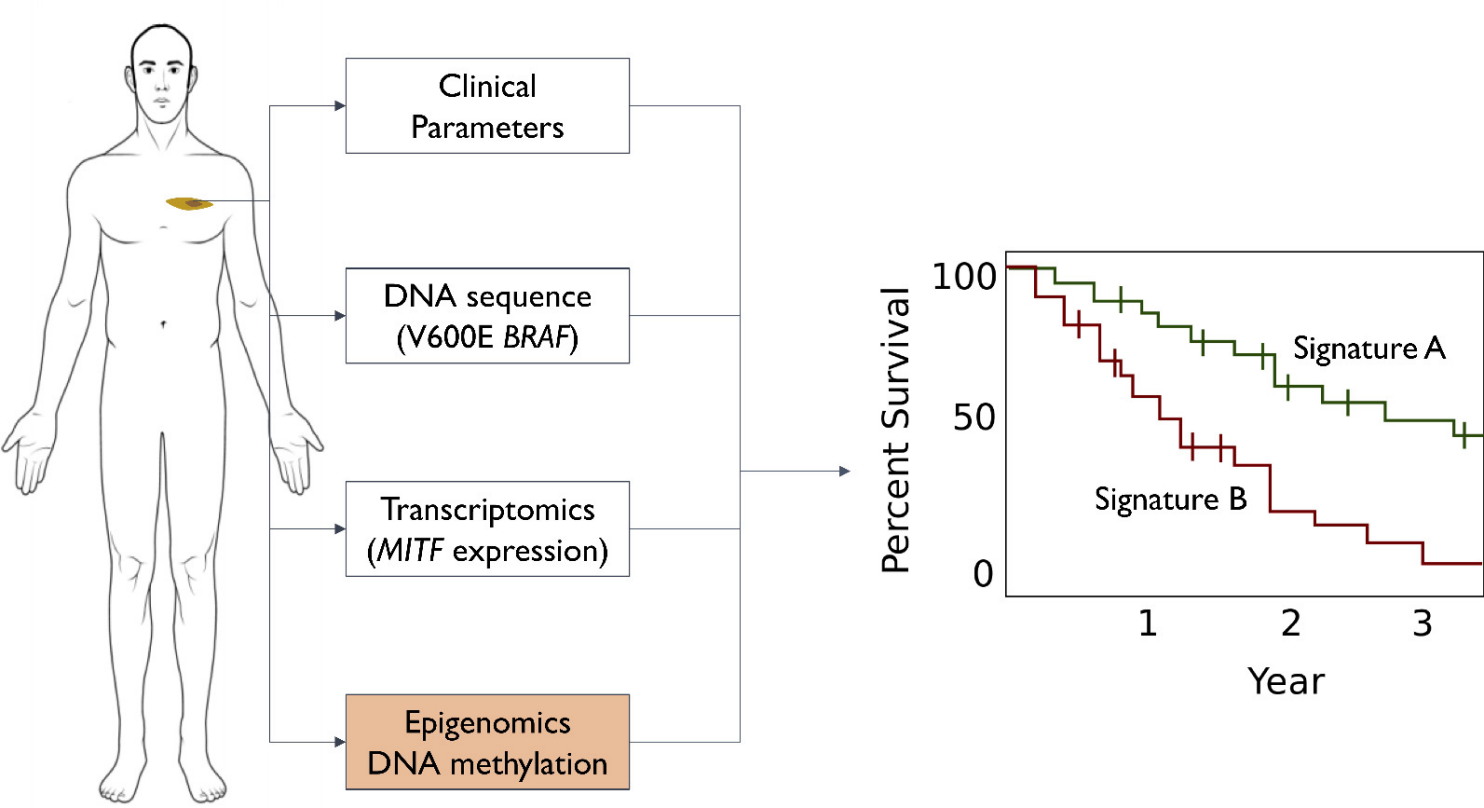
Different ways to predict survival rates for patients with melanoma. Some patients with a given cancer have higher survival rates than other patients with the same type of cancer: the discovery of signatures for higher (green line in graph) or lower (red line) survival rates would help doctors to manage the expectations of their patients. Survival predictions for cutaneous melanoma were originally based on clinical parameters: tumor location, Breslow thickness (how deep it spreads into the skin), stage (size and distance spread), and grade (how its cells look under the microscope). Advances in cancer genetics led to the discovery of biomarkers (such as the V600E mutation in the *BRAF* gene) that enabled more accurate predictions. Advances in transcriptomics also led to biomarkers, such as the level of transcription of a gene called *MITF*. Guo et al. complemented these approaches by analyzing epigenomics data to identify a biomarker based on DNA methylation marks (orange box): the predictive power of the new biomarkers is higher than that of previous biomarkers.

IMAGE CREDIT:Rw251 [CC0 1.0].

To continue the search for better biomarkers researchers went from studying genomics and transcriptomics to studying epigenomic changes such as DNA methylation (Figure 1). Multiple studies have shown that the addition of methyl group to certain DNA nucleotides plays important roles in tumor formation and cancer progression. Furthermore, these methyl markers are easily detectable and remain stable in biological samples, making them clinically useful as biomarkers (Keeley et al., 2013). Now, in eLife, Qiang Wang, Jian-Qun Chen and co-workers at Nanjing University and Shanghai University – including Wenna Guo and Liucun Zhu as joint first authors – report the discovery of a biomarker based on DNA methylation that provides the most accurate predictions of melanoma survival to date (Guo et al., 2019).

Guo et al. studied the methylation profile of 461 cutaneous melanoma patients from the Cancer Genome Atlas Project (International Cancer Genome Consortium et al., 2010). Regression analysis of this dataset revealed 4,454 DNA methylation sites that were associated with overall melanoma survival. Exploring all possible combinations of these markers identified a combination of four methylation marks that could optimally predict the survival of melanoma patients (Figure 1). Intriguingly, two out of the four methylation marks are in close proximity to two genes that are known to be associated with cutaneous melanoma: *OCA2,* which was found to be genetically varied in melanoma patients (Law et al., 2015), and *RAB37*, which is a member of an oncogene family.

Understanding the biological basis of the link between these methylation marks and survival will be challenging. DNA methylation could be controlling gene expression: however, the direction of this effect would need to be determined on gene by gene basis. Interestingly, Guo et al. also found that their four-methylation-mark signature has similarities to a signature used in cancer immunotherapy. The predictive power of the new biomarker is also higher than that of other biomarkers, including the five-DNA methylation signature that can predict the immune response to tumors (Jeschke et al., 2017).

Improvements in our ability to predict disease outcome are valuable in their own right. Moreover, a better understanding of the biology responsible for the correlations observed between the methylation signature, gene expression and immunotherapy targets has the potential to contribute to the global efforts to find a cure for melanoma.
